# Parental Bonding, Emotion Regulation, and Addiction Severity in Alcohol Use Disorder

**DOI:** 10.1192/j.eurpsy.2026.10502

**Published:** 2026-07-31

**Authors:** D. D. Demir, M. Ergelen

**Affiliations:** 1 Newham Early Intervention Service, East London NHS Foundation Trust, London, United Kingdom; 2 Psychiatry, Erenkoy Mental and Nervous Diseases Training and Research Hospital, Istanbul, Türkiye

## Abstract

**Introduction:**

Alcohol use is known to be associated with the self-medication hypothesis as a maladaptive coping mechanism of individuals who have difficulties in emotion regulation. The capacity for emotion regulation develops in line with the bonding characteristics with the caregivers in the early stages of development.

**Objectives:**

This study aims to explore the relationship between parental bonding and addiction severity in Alcohol Use Disorder (AUD), with a focus on the mediating role of emotion regulation difficulties.

**Methods:**

A total of 210 male participants (105 diagnosed with AUD, 105 healthy controls) aged 18–65 were recruited from outpatient and inpatient AUD units. Data were collected using the Sociodemographic and Clinical Data Form, the Parental Bonding Inventory (PBI), the Difficulties in Emotion Regulation Scale (DERS), and the Addiction Profile Index (API). Parental bonding, emotion regulation difficulties, and addiction severity were assessed through the PBI, DERS, and API, respectively. Parametric tests (t-test, ANOVA), regression, and mediation analyses (Hayes & Preacher, 2004, PROCESS in SPSS) were conducted to examine group differences, predictors, and mediating effects related to the severity of alcohol addiction.

**Results:**

Statistical analysis revealed that the AUD group had lower scores on the PBI Protection subscale for both mothers and fathers compared to the control group (p<0.05), and higher scores on the DERS Nonacceptance, Goals, Impulse, Strategy, Clarity, and Total scores (p<0.05). Mother and father PBI Care/Control and Total scores were negatively associated with API Substance Use Characteristics (p<0.05). API Total score was positively associated with DERS Nonacceptance, Goals, Impulse, Strategy, Clarity, and Total scores (p<0.05). A full mediating effect of DERS Total score was found in the relationship between mother’s PBI Care/Control, mother and father’s PBI Total scores, and API Substance Use Characteristics (p<0.05). A partial mediating effect of DERS Total score was found in the relationship between father’s PBI Care/Control and API Substance Use Characteristics (p<0.05).

**Conclusions:**

The findings suggest that AUD patients reported more overprotective parental bonding styles during childhood and adolescence compared to healthy controls. Difficulties in emotion regulation were also more severe in the AUD group. Higher levels of parental care and control were associated with lower severity of alcohol addiction, with emotion regulation difficulties mediating this relationship.These results indicate that early bonding experiences and the development of emotion regulation strategies may play a crucial role in both assessing risk and predicting the course of AUD. Based on these findings, treatment approaches that target early relational patterns and emotion regulation, such as Cognitive Analytic Therapy, which emphasises early caregiver experiences, may provide promising treatment options.

**Disclosure of Interest:**

None Declared

